# Differential Genomic Imprinting and Expression of Imprinted microRNAs in Testes-Derived Male Germ-Line Stem Cells in Mouse

**DOI:** 10.1371/journal.pone.0022481

**Published:** 2011-07-22

**Authors:** Ji Young Shin, Mukesh Kumar Gupta, Yoon Hee Jung, Sang Jun Uhm, Hoon Taek Lee

**Affiliations:** Department of Animal Biotechnology, Bio-Organ Research Center/Animal Resources Research Center, Konkuk University, Hwayang-dong, Gwangjin-gu, Seoul, South Korea; City of Hope National Medical Center and Beckman Research Institute, United States of America

## Abstract

**Background:**

Testis-derived male germ-line stem (GS) cells, the in vitro counterpart of spermatogonial stem cells (SSC), can acquire multipotency under appropriate culture conditions to become multipotent adult germ-line stem (maGS) cells, which upon testicular transplantation, produce teratoma instead of initiating spermatogenesis. Consequently, a molecular marker that can distinguish GS cells from maGS cells would be of potential value in both clinical and experimental research settings.

**Methods and Findings:**

Using mouse as a model system, here we show that, similar to sperm, expression of imprinted and paternally expressed miRNAs (miR-296-3p, miR-296-5p, miR-483) were consistently higher (P<0.001), while those of imprinted and maternally expressed miRNA (miR-127, miR-127-5p) were consistently lower (P<0.001) in GS cells than in control embryonic stem (ES) cells. DNA methylation analyses of imprinting control regions (ICR), that control the expression of all imprinted miRNAs in respective gene clusters (*Gnas-Nespas* DMR, *Igf2-H19* ICR and *Dlk1-Dio3* IG-DMR), confirmed that imprinted miRNAs were androgenetic in GS cells. On the other hand, DNA methylation of imprinted miRNA genes in maGS cells resembled those of ES cells but the expression pattern of the imprinted miRNAs was intermediate between those of GS and ES cells. The expression of imprinted miRNAs in GS and maGS cells were also altered during their in vitro differentiation and varied both with the differentiation stage and the miRNA.

**Conclusions:**

Our data suggest that GS cells have androgenetic DNA methylation and expression of imprinted miRNAs which changes to ES cell-like pattern upon their conversion to maGS cells. Differential genomic imprinting of imprinted miRNAs may thus, serve as epigenetic miRNA signature or molecular marker to distinguish GS cells from maGS cells.

## Introduction

Germ-line stem (GS) cells, the *in vitro* counterpart of spermatogonial stem cells (SSC) in the testis, can self-renew *in vitro* for more than two years and, when transplanted into the seminiferous tubules of an infertile male mouse, can establish donor-derived spermatogenesis to transmit the donor haplotype to progeny [1], [2], [3], [4]. Upon extended *in vitro* culture, a second cell type, called multipotent GS (mGS; from neonatal testes) or multipotent adult GS (maGS; from adult testes) cells, also appears from GS cells that can be expanded selectively under culture conditions used for embryonic stem (ES) cells. Unlike GS cells, mGS or maGS cells show multipotency and produce teratoma upon transplantation into the seminiferous tubules of the recipient testis [5], [6]. The mGS and maGS cells originate from the cultured GS cells themselves at a low frequency and are not some leftover of earlier type of germ cells [1], [6], [7], [8]. During this conversion, the androgenetic genomic imprinting in GS cells also changes to ES cell-like pattern in mGS or maGS cells [6], [9]. Recently, we showed that mouse maGS cells are epigenetically stable for DNA methylation at imprinted *Igf2-H19* gene cluster during *in vitro* culture and differentiation [10] but re-acquire GS cell-like growth and differentiation characteristics with altered DNA methylation pattern when they are re-cultured in the GS-like conditions [11]. Thus, at any particular time point, *in vitro* cultured GS cells may contain some contaminating mGS or maGS cells which may produce teratoma instead of initiating spermatogenesis upon their transplantation into recipient testis [5], [6], [11]. Consequently, a molecular marker that can distinguish GS cells from mGS or maGS cells would be of potential value in both clinical and experimental research settings.

MicroRNA (miRNA) are a class of 20–25 nucleotide-long non-coding endogenous RNAs that post-transcriptionally modulate the gene expression through canonical base pairing between the seed sequence of the miRNA (nucleotides 2–8 at its 5′ end) and its complementary seed match sequence in the 3′UTR of target mRNAs [12]. Imprinted miRNAs represent a family of miRNA that are mono-allelically expressed in a parent-of-origin manner and act in trans, generally outside the genomic region from where they arise [13], [14]. Genes encoding the imprinted miRNAs are mainly clustered in two chromosomal domains [PWS-AS (also called *Snurf-Snrpn*) cluster and *Dkl1-Gtl2* cluster] in mouse although few single imprinted miRNA are also present at several genomic regions [14], [15], [16], [17], [18], [19]. Furthermore, almost all well-characterized imprinted genes clusters such as *Igf2-H19*, *Peg10*, *Copg2*, *Rasgfr1*, *Gnas-Nespas*, *Kcnq1* and *Igf2r-Air* also encode one or more imprinted miRNAs whose expression is restricted in a parent-of-origin manner and is controlled by DNA methylation at imprinting control region (ICR) of the respective gene cluster [14], [15], [16]. These imprinted miRNAs show distinct temporal- and tissue-specific expression patterns in different tissues, including ES cells, and control a wide range of developmental and physiological pathways, including stem cell pluripotency and differentiation [20], [21], [22], [23], [24], [25], [26]. Recent studies have shown that maGS and ES cells have similar miRNA profile [27], [28]. However, miRNA profile of GS cell has not been investigated. We recently showed that GS and maGS cells show differential expression of Let-7 and miR-294 miRNAs which may serve as miRNA signature to distinguish GS cells from maGS or ES cells [29]. However, nothing is known in literature about genomic imprinting or expression of imprinted miRNA in GS and maGS cells. Recent studies have also shown that, a cluster of imprinted miRNA encoded by imprinted *Dlk1-Dio3* locus correlates with the pluripotency levels of mouse stem cells [22] and consequently, imprinting status of this locus can serve as a marker to identify fully pluripotent iPS (induced pluripotent stem cell) or ES cells from partial pluripotent cells [22], [25]. However, genomic imprinting and expression of imprinted miRNAs, including those encoded by *Dlk1-Dio3* locus, in testes-derived male germ-line stem cells are not known.

**Figure 1:**
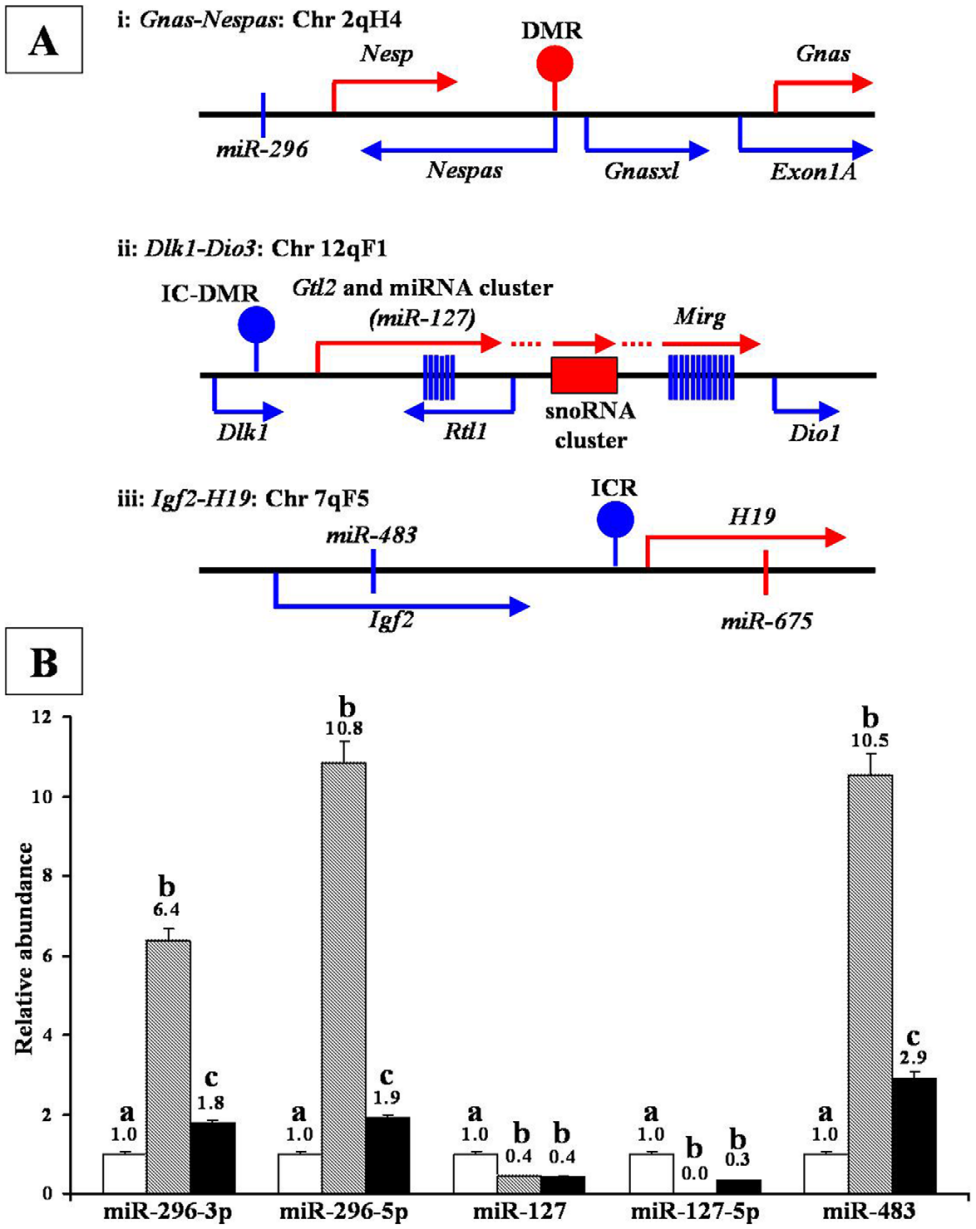
Genomic location and expression of imprinted miRNAs analyzed in this study. A: Genomic structure of *Gnas-Nespas* (i), *Dlk1-Dio3* (ii) and *Igf2-H19* (iii) imprinted gene clusters analyzed in this study. Maternally expressed genes and miRNAs are shown in red while paternally expressed genes and miRNAs are shown in blue. Arrow shows the direction of transcription. Lollipops represent the position of imprinting control region (ICR) or differentially methylated regions (DMR) of the respective gene cluster. Red lollipop indicates methylation in maternal chromosome while blue lollipops indicated methylation in paternal chromosomes. Not drawn to scale. B: Expression of miR-296-3p, miR-296-5p, miR-127, miR-127-5p and miR-483 imprinted miRNAs in mouse somatic cells (open box), sperm (crossed box) and testis-tissue (closed box). Values above the bars indicate relative abundance of miRNAs normalized to the expression of respective miRNA in somatic cells. Different alphabet (a, b, c) on the bars indicate statistical difference (P<0.01) in gene expression.

Therefore, we extended our earlier study [29], to investigate the genomic imprinting and expression of a selected number of imprinted miRNAs in mouse testes-derived male germ-line stem cells. Three imprinted miRNAs, miR-127, miR-483 and miR-296, which are encoded from *Dlk1-Dio3*, *Igf2-H19* and *Gnas-Nespas* imprinted gene clusters, respectively under the regulation of common ICRs (*Dlk1-Dio3* IG-DMR, *Igf2-H19* ICR and *Gnas-Nespas* DMR) for all miRNAs in the respective gene clusters were analyzed (Figure 1A) [20], [30], [31], [32]. These imprinted gene clusters were specifically chosen because DNA methylation at both *Dlk1-Dio3* IG-DMR and *Igf2-H19* ICR is imprinted (i.e., DNA methylated) on paternal chromosome to suppress the expression of miR-127 and miR-483 from paternal and maternal chromosome, respectively. On the other hand, *Gnas-Nespas* DMR is imprinted (i.e., DNA methylated) on maternal chromosome to suppress the expression of miR-296 from maternal chromosome [14], [15], [30]. Thus, analysis at these three imprinted gene clusters encompass all possible combinations of paternal imprinting controlling maternally- or paternally- expressed miRNAs and *vice versa*.

## Materials and Methods

All chemicals were obtained from Sigma-Aldrich Co. (St. Louis, MO, USA) unless otherwise specifically indicated. All animal experimentations of the study were approved by the Institutional Bio-safety Committee (Approval ID Nos. : 09-01-020, 09-01-022, 09-01-025, 09-01-026, and 09-01-027) that oversees the ethical and animal experimental issues.

### Establishment of GS and maGS cell lines

The GS and maGS cells were isolated from 4- to 6- week old DBA/2 mouse (Orient Bio, South Korea) testes as described earlier with partial modifications [5], [6], [29]. The isolated cells were cultured on mitomycin C- treated STO feeder cell layer in Dulbecco's modified Eagle medium (DMEM) supplemented with 15% (v/v) fetal bovine serum (Hyclone, Logan, UT), MEM nonessential amino acids (Gibco BRL, Grand Island, NY), 1% penicillin-streptomycin (Gibco BRL), 50 µM 2-mercaptoethanol, and 10 ng/ml recombinant human GDNF (R & D Systems, Minneapolis, MN) at 37°C in a humidified atmosphere of 5% CO_2_ in air. Additionally, 1000 units/ml murine leukemia inhibiting factor (LIF; Chemicon, Temecula, CA) was added to initial culture medium to enhance the formation of germ cell colonies [33]. The GS and maGS cell colonies were subsequently cultured in the absence (GS) or presence (maGS) of LIF to establish the respective cell lines [5], [6]. The established maGS cell lines were subsequently maintained in feeder-free conditions while GS cell lines were cultured on mitomycin C- treated STO feeder cell layer. The characteristics of established GS and maGS cell lines were verified by expression of marker genes and alkaline phosphatase (AP) activity essentially as we described previously [10], [11] (Supplementary Figure S1). The maGS cells, cultured under the current culture conditions, resembled the ES cells for the hemi-zygotic DNA methylation pattern of *Igf2-H19* ICR and the expression of Let-7 and miR-294 miRNAs (Supplementary Figure S2).

For *in vitro* differentiation studies, GS and maGS cells were induced to form embryoid bodies (EB) by hanging drop culture for four days and then cultured in the presence of 1.0 µM all-trans retinoic acid (EBRA) for next four days as we described earlier [10], [11]. As control, mouse ES cells were cultured for analysis by standard method [34].

### Analyses of miRNAs

The expression of miRNAs was quantified by real-time TaqMan MicroRNA Assay essentially as we described earlier [29]. Briefly, total RNA, including miRNA, was extracted from cultured cells using the mirVana^R^ microRNA isolation kit (Ambion, Austin, TX, USA) according to the manufacturer's instructions. The isolated miRNAs (10 ng) were then converted to cDNA using TaqMan^R^ MicroRNA Reverse Transcription Kit (Ambion) in 15 µl reaction mixture containing 100 mM dNTPs, 50 U/µl MultiScribe^R^ reverse transcriptase, 20 U/µl RNase inhibitor and 3 µl of miRNA-specific reverse transcription (RT) primer according to the manufacturer's instructions and amplified for specific miRNA on a 7500 Fast Real-Time PCR System (Applied Biosystems, Foster City, CA) using commercially available optimized miRNA-specific primers for each miRNA and for the endogenous sno202 control. The amplification parameters for RT and real-time PCR were employed according to the manufacturer's protocol (TaqMan^R^ MicroRNA assay kit; Applied Biosystems). This method quantifies exclusively mature miRNAs but not their precursors. The expression values for each miRNAs were normalized to endogenous sno202 control and relative expression values were obtained using the 2^−ΔΔCt^ method (Applied Biosystems user bulletin PN 4347825). All experiments were performed in triplicates.

### Analyses of DNA methylation

DNA methylation status of GS and maGS cells was analyzed by bisulfite genomic sequencing essentially as we described earlier [10], [11]. Briefly, genomic DNA (2 µg) was treated with sodium bisulfite using Epitect bisulfite kit (Qiagen, Hilden, Germany) and PCR-amplified using specific primer pairs for *Dlk1-Dio3* IG-DMR, *H19* ICR and *Gnas-Nespas* DMR (Supplementary Table S1). Amplified PCR-products were then cloned in pGEM-T Easy vector (Promega, Madison, WI, USA), sequenced in 3730 DNA Analyzer (Applied Biosystems) and analyzed for methylation status using BiQ Analyzer [35]. As control, genomic DNA from mouse sperm and oocytes were also analyzed.

### Analyses of gene expression

Gene expression in GS and maGS cells were analyzed by semi-quantitative RT-PCR or real time quantitative RT-PCR (qRT-PCR) essentially as we described earlier [10], [11]. The primer pairs used for PCR amplification are shown in Supplementary Table S2.

### Statistical analyses

The expression levels of genes and miRNAs were compared by ANOVA using SPSS software (SPSS Inc, Chicago, IL, USA). Differences at P≤0.05 were considered significant.

## Results and Discussion

**Figure 2:**
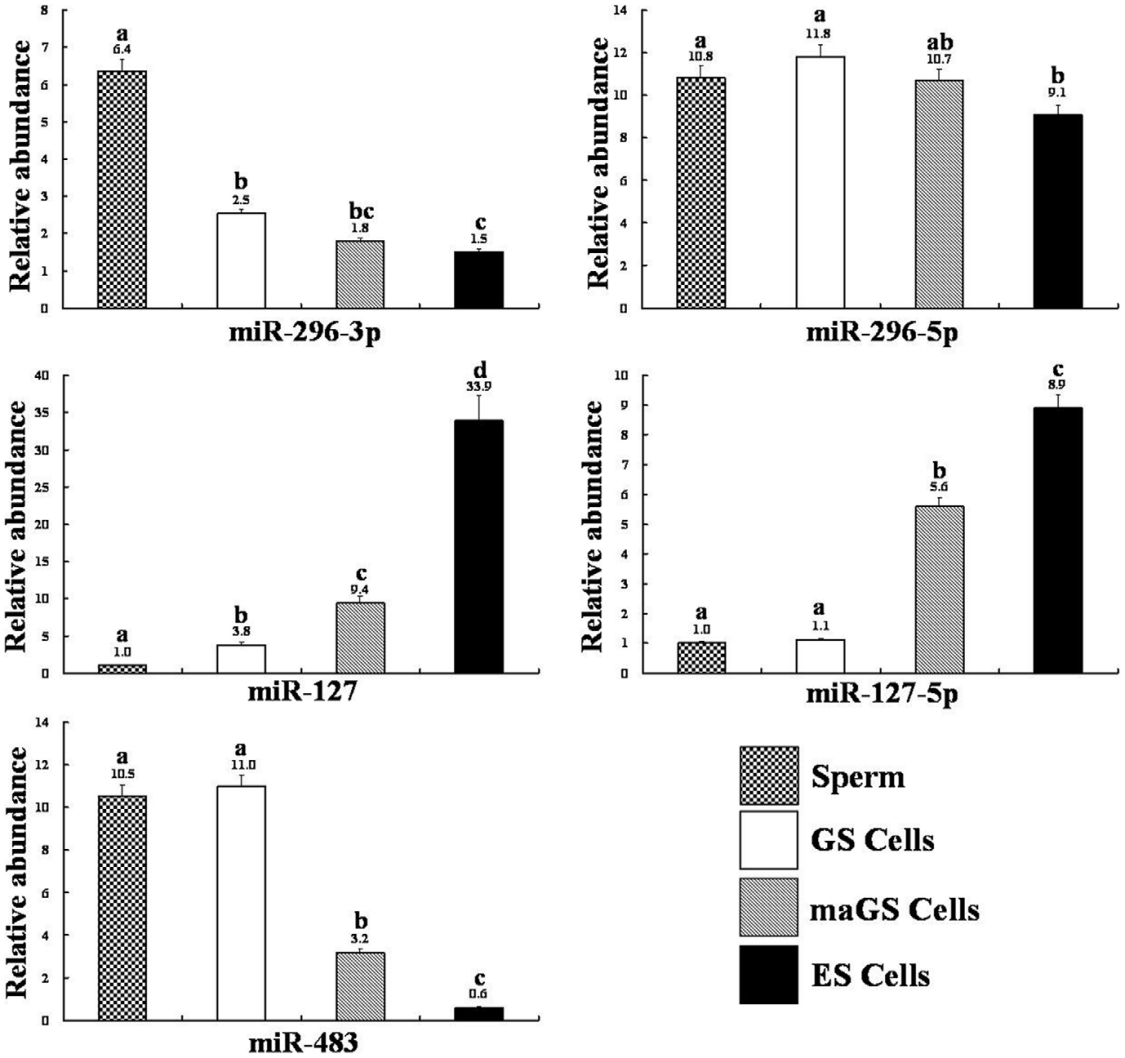
Expressions of miR-296-3p, miR-296-5p, miR-127, miR-127-5p and miR-483 imprinted miRNAs in male germ-line (GS) and multipotent adult germ-line (maGS) stem cells. Sperm and Embryonic stem (ES) cells were used as controls for comparison. All data were normalized to endogenous sno202 RNA as internal controls and calibrated on the STO cells, whose expression was considered one for all genes except for the miR-127 and miR-5p, for which sperm were used as a calibrator and considered one. Values above the bars indicate relative abundance of respective miRNAs. Different alphabet (a, b, c. d) on the bars indicate statistical difference (P<0.01) in gene expression.

**Figure 3:**
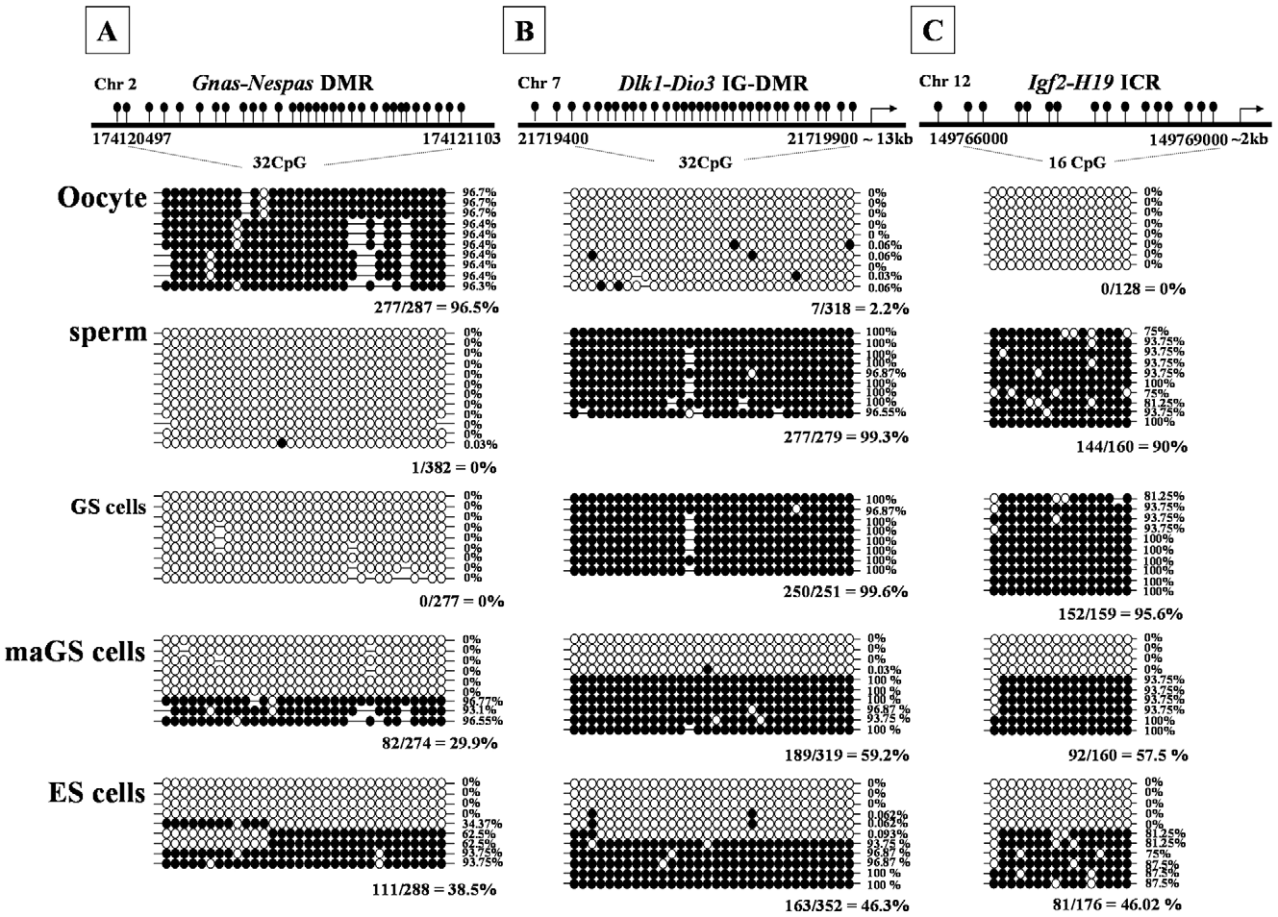
DNA methylation status of imprinting control regions that control the expression of imprinted miRNAs at *Gnas-Nespas* (*Gnas-Nespas* DMR), *Dlk1-Dio3* (*Dlk-Dio3* IG-DMR) and *Igf2-H19* (*Igf2-H19* ICR) loci in male germ-line (GS) and multipotent adult germ-line (maGS) stem cells. Sperm, oocyte and embryonic stem (ES) cells were used as control for comparison. Each line represents a separate clone and each CpG site is shown by its sequence position in the GenBank sequence. Each circle indicates individual CpG residues within the areas amplified. Open and closed circles indicate unmethylated and methylated CpGs, respectively.

We observed that the expression pattern of imprinted miRNAs in mouse sperm was distinct from those of ES or somatic cells (Figure 1B). Sperm showed significantly higher (P<0.001) expression of imprinted and paternally expressed miRNAs (miR-296-3p, miR-296-5p, miR-483) and lower (P<0.001) expression of imprinted and maternally expressed miRNAs (miR-127, miR-127-5p) than those observed with somatic cells (Figure 1B). Similar expression patterns of imprinted miRNAs were also observed in testicular tissue although the deviation in miRNA levels, from those of somatic cells, was less prominent than those observed with sperm, possibly due to the presence of both somatic and germ cells in the testicular tissue. Similar to sperm, the expression of imprinted and paternally expressed miRNAs (miR-296-3p, miR-296-5p, miR-483) were consistently higher (P<0.001) while those of imprinted and maternally expressed miRNA (miR-127, miR-127-5p) were consistently lower (P<0.001) in GS cells than in control ES cells (Figure 2). These data suggest that the expression pattern of imprinted miRNA in mouse GS cells is likely androgenetic and therefore, may possibly form an epigenetic signature on testes-derived male germ-line stem cells. To further confirm this, we performed DNA methylation analysis of ICRs that control the expression of all imprinted miRNAs in respective gene clusters [16], [20], [30], [31], [32], [36]. We found that, 0.0, 99.6 and 95.0% of CpGs in *Gnas-Nespas* DMR, *Igf2-H19* ICR and *Dlk1-Dio3* IG-DMR, respectively were methylated in GS cells and were similar to 0.0, 99.3 and 90.0% methylation observed for respective sites in sperm (Figure 3). Thus, genomic imprinting of imprinted miRNAs is androgenetic in GS cells.

On the other hand, maGS cells resembled ES cells for the DNA methylation of imprinted miRNA genes as has also been reported earlier for several other imprinted genes [6], [9]. However, expression pattern of the imprinted miRNAs in maGS cells was intermediate between GS and ES cells and did not completely resemble the ES cell-like expression pattern (Figure 2). Similar results were also observed for the expression pattern of mRNA transcripts encoded by these imprinted gene clusters (Supplementary Figure S3) except for the *Igf2-H19* as we had shown previously [10], [11], [29]. These data confirm the conversion of androgenetic GS cells to multipotent maGS cells [1], [6], [7], [11] but also suggest that the acquisition of ES cell-like characteristics in maGS cells was likely incomplete or partial [6], [7], [11]. Indeed, previous studies have also shown that genomic imprinting in maGS cells stand in between GS and ES cells [6], [7]. The maGS cells also expressed higher level of germ cell markers characteristic of primordial germ cells and spermatogonia compared with ES cells [28], although the expression pattern of the ES cell-specific miR-290 and miR-302 cluster of miRNA in maGS cells resembled that of the ES cells [27]. Thus, in case of maGS cells, which show discrepancy between DNA methylation and transcription pattern of imprinted miRNAs, it appears that expression of individual imprinted miRNAs may be leaky [37], [38] or may be modified by in vitro manipulation, physiological state, level of differentiation, in vitro culture conditions or physiological function of the miRNA [11], [22], [25], [29], [37]. The DNA methylation and expression of imprinted miRNA in maGS cells may also be modified by the level of reprogramming [22], [25]. In particular, imprinting status of the imprinted miRNAs in the *Dlk1-Dio3* locus was shown to correlate with the pluripotency levels of the mouse stem cells [22], [25]. However, we found that, unlike partially reprogrammed iPS cells, mouse maGS cells did not show aberrant DNA methylation at *Dlk1-Dio3* IG-DMR and had hemi-zygotic methylation pattern (59.2% vs. 46.3%) of ES cells. Ability to generate partial, incomplete and fully reprogrammed maGS cells in future may help determine, if imprinting status of *Dlk1-Dio3* IG-DMR could be an indicator of reprogramming in maGS cells, as has been reported earlier for the ES cells [22], [25]. The difference in DNA methylation and miRNA expression was not due to difference in their chromosomal composition as both GS, maGS and ES cells, used in this study, were of male origin and contained XY sex chromosomes.

**Figure 4:**
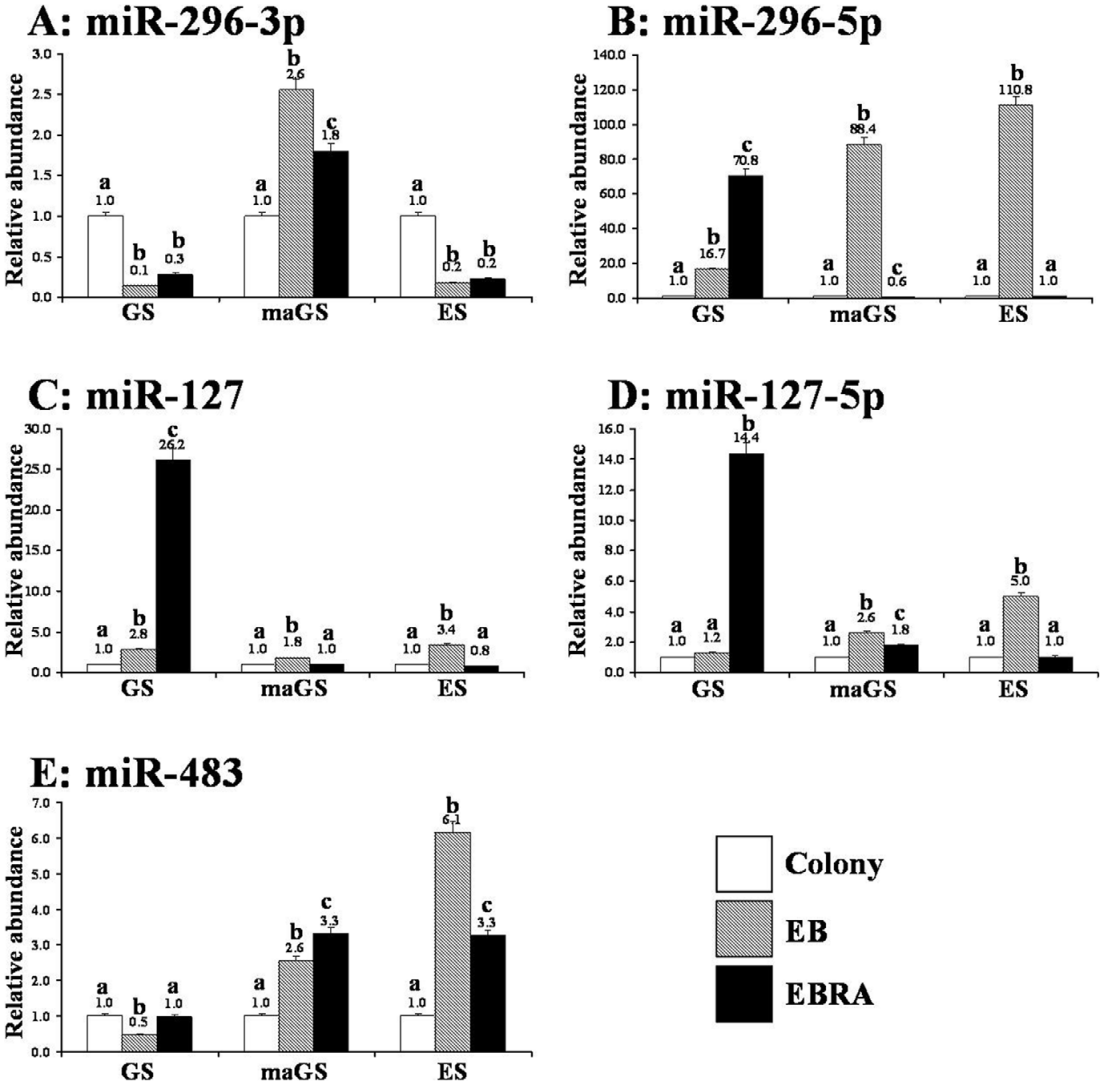
Expression of miR-296-3p (A), miR-296-5p (B), miR-127 (C), miR-127-5p (D) and miR-483 (E) imprinted miRNAs during in vitro differentiation of male germ-line (GS) and multipotent adult germ-line (maGS) stem cells. Embryonic stem (ES) cells were used as control for comparison. Undifferentiated colonies were induced to form embryoid bodies (EB) by hanging drop method for 4 days and further treated with all-trans retinoic acid (EBRA) for another 4 days. Values above the bars indicate relative abundance of miRNAs normalized to sno202 RNA as internal controls and calibrated on the expression of respective miRNA in undifferentiated colonies of respective group. Different alphabet (a, b, c) on the bars indicate statistical difference (P<0.01) in gene expression within GS, maGS or ES cell group.

We also evaluated whether expression of imprinted miRNAs in mouse GS and maGS cells alters during their *in vitro* differentiation. As expected, stem cell (*Oct4*, *Nanog*, *Cd9*, *Rex1*), and GS cell (*Stra8*, *Cd9*) marker genes were silenced during differentiation of GS and maGS cells but was not complete in ES cells (Supplementary Figure S1). During differentiation, expression of imprinted miRNAs also underwent changes but varied both with the differentiation stage and the miRNA (Figure 4). Given that the EBs contain a mixed population of cells from three germ layers [39] and that the expression of individual imprinted miRNA may vary with the cell/tissue types wherein they may have different functions [14], [15], [20], [21], [23], [24], [26], the differential expression pattern of imprinted miRNAs in differentiating GS, maGS and ES cells may reflect their differential ability to differentiate into various cell types [11], [40], [41], [42], [43]. Interestingly, although a clear pattern was not evident, changes in the expression pattern of imprinted miRNA during in vitro differentiation of maGS cells were apparently more similar to those of ES cells and clearly differed from differentiating GS cells (Figure 4). This observation is similar to those of Zovoilis et al., [28], [44] who found that maGS cells resembled ES cells, but the expression of certain miRNAs such as miR-290 cluster was retained during their in vitro differentiation. We also observed that, similar to previous reports on several miRNAs [45], [46], mature miRNA originating from both 3′ (miR-296-3p) and 5′ (miR-296-5p) arms of the miR-296 accumulated as sister pairs in undifferentiated testis-derived germ-line stem cells (Figure 1B). However, their expression pattern differed among the differentiating cells of the three groups (Figure 4A and 4B) and, the EBs generated from GS cells resembled those of ES cells for the expression pattern of miR-296-3p. Since the phenomenon of miRNA strand selection for the functional stability (-3p vs. -5p vs. both) occurs in a tissue-dependent manner [45], [46], [47], the differences in the expression of miR-296-3p and miR-296-5p among the EBs of the three groups probably reflects the different proportion of cells of three germ-layers in them [11], [39], [40], [41].

It was also observed that, differentiating EBs generated from GS cells had significantly high level of miR-127 and miR-127-5p (Figure 4C and 4D), which might suggest their possible role during in vitro differentiation of SSC. Our result is similar to a previous study which showed high expression of miR-127 in testicular samples [46]. The miR-127 was preferentially expressed in immature mouse testes that principally contained mitotically active spermatogonia (day 7), meiosis I spermatocyte (day 12) and round spermatid (day 21), but remained at medium level in purified pachytene spermatocyte and round spermatid and declined in adult testis (containing fully differentiated germ cells and spermatozoa) [46]. However, the mechanism by which miR-27 affects the SSC biology remains unclear. It is likely that miR-127 may act by down-regulating its target proteins such as *Bcl6* [48] and *Brd2* [49] which are important for GS cell self-renewal and for the inhibition of differentiation, respectively [50], [51], [52], [53]. The *Brd2* was also shown to be expressed at high levels in diplotene spermatocytes and round spermatids and at low levels in spermatogonia that negatively co-related with the expression pattern of miR-127 [49], [53].

**Figure 5:**
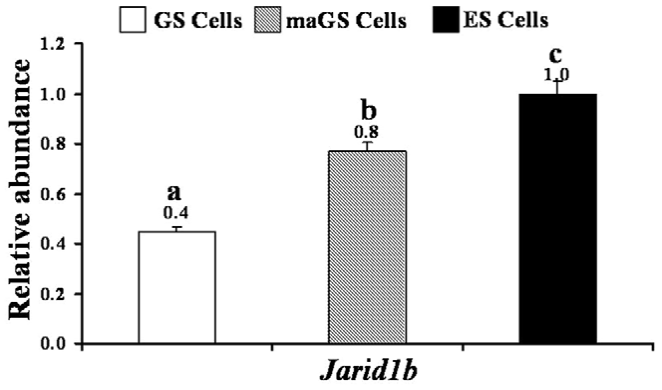
Expression of Jarid1b, a putative target of miR-483 miRNA in male germ-line (GS) and multipotent adult germ-line (maGS) stem cells. Embryonic stem (ES) cells were used as control for comparison. Values above the bars indicate relative abundance of mRNAs normalized to the expression of respective mRNA in undifferentiated ES cells. Bars with different alphabet (a, b, c) differ significantly (P<0.01).

The functions of imprinted miRNAs in testis-derived male germ-line stem cells are not known. They acts in trans, generally outside the genomic region from where they arise [13], [14], and may even cleave the mRNAs encoded by the same imprinted gene cluster in partner chromosome (for example, maternally expressed miRNAs can silence a paternally expressed gene or vice versa) in a RNAi-like manner [23], [54], [55]. Liu et al., [22] found that imprinted miRNAs encoded by *Dlk1-Dio3* locus (e.g. miR-127 and miR-127-5p) had 717 putative targets that were related to multiple aspects of growth, differentiation, metabolism and other developmental processes in pluripotent cells. Furthermore, several miRNAs from this cluster potentially target the PRC2 silencing complex to form a feedback regulatory loop resulting in the expression of all genes and non-coding RNAs encoded by this locus [22]. On the other hand, *Gnas-Nespas* cluster encode miR-296 and miR-298 which are derived from non-coding *Nespas* gene transcript. The miR-296 regulates the expression of growth factor receptor in endothelial cells [56] and increases upon in vitro differentiation of ES cells to target the *Nanog* gene transcript [57]. The *Igf2-H19* cluster encodes miR-675 and miR-483 but their precise role in stem cells is not known. An *in silico* bioinformatic analysis using web-based TargetScan (URL: http://www.targetscan.org/mmu_50/) and MicroCosm Targets Version 5 (URL: http://www.ebi.ac.uk/enright-srv/microcosm/htdocs/targets/v5/) softwares showed that miR-483 has numerous putative targets (∼975 in mouse and ∼1072 in human) that included *Jarid1b* (data not shown). *Jarid1b* (Histone demethylase KDM5b) directly regulates genes that control cancer cell proliferation [58] and may be essential for the stem cell pluripotency [57], [59]. Although *Jarid1b* is yet to be validated as a target of miR-483, we found that consistent with the high expression of miR-483 in GS cells, the expression of *Jarid1b* was significantly lower in GS cells than in maGS or ES cells (Figure 5). Moreover, consistent with the expression of *Jarid1b*, GS cells proliferated slower than maGS cells (∼4–6 days for GS cells and ∼2–3 days for maGS cells), as has also been reported earlier [11], [60].

In conclusion, our data suggest that genomic imprinting and expression of imprinted miRNAs are androgenetic in mouse GS cells but changes to ES cell-like pattern upon their conversion to maGS cells. Differential genomic imprinting of imprinted miRNAs may thus serve as epigenetic signature or molecular marker to distinguish GS cells from maGS or ES cells. Since maGS cells originate from GS cells during their extended in vitro culture [1], [5], [6], [7], [8], [11], our data may have implications in clinical settings to distinguish GS cell colonies from maGS cell colonies and thereby minimize the likelihood of teratoma formation by contaminating maGS cells generated from the GS cells. Conversely, in experimental research settings and regenerative medicine, analysis of imprinted miRNA may help in discriminating maGS cells from GS cells for tissue engineering or studying cellular reprogramming. Prior to clinical and/or research applications, putative GS/maGS colonies may be screened for imprinting status of imprinted miRNAs using sperm and ES cells as controls. An androgenetic imprinting status comparable to those of sperm would suggest that the colony originated from GS cells. However, it must be noted that imprinted miRNA is not a marker of stemness in the testes-derived male germ-line stem cells and therefore, may not be used as a tool for their isolation and/or identification.

## Supporting Information

Figure S1 **Characterization and in vitro differentiation of male germ-line (GS) and multipotent adult germ-line (maGS) stem cells in mouse.** Embryonic stem (ES) cells were used as control for comparison. A: Colony characteristic, alkaline phosphatase (AP) activity and embryoid bodies generated from GS, maGS and ES cells. Calibration bar: 100 µ. B: Expression of stem cell and germ-cell marker genes in undifferentiated colonies, embryoid bodies (EB) and all-trans retinoic acid -treated embryoid bodies (EBRA) of GS, maGS and ES cells.
(TIF)

Figure S2 **DNA methylation status of** ***Igf2-H19*** **gene cluster (A) and expression of Let-7a, Let-7d and miR-194 miRNAs (B) in multipotent adult germ-line (maGS) stem cells cultured in two different culture conditions.** maGS (1): maGS cells cultured in the presence of GDNF, LIF and STO feeder cell; maGS (2): maGS cells cultured in feeder-free ES cell-like culture condition. Embryonic stem (ES; open box) cells were used as controls for comparison. Values above the bars indicate relative abundance of miRNAs normalized to the expression of snoRNA in respective cells and calibrated on undifferentiated ES cells. Different alphabet (a, b, c) on the bars indicate statistical difference (P<0.01) in respective gene expression.
(TIF)

Figure S3 **Expression of imprinted genes encoded by** ***Gnas-Nespas*** **(** ***Gnas*** **and** ***Gnasxl*** **),** ***Dlk1-Dio3*** **(** ***Dlk1*** **and** ***Meg3*** **) an** ***Igf2-H19*** **(** ***H19*** **and** ***Igf2*** **) gene clusters in male germ-line (GS; open box) and multipotent adult germ-line (maGS; crossed box) stem cells.** Embryonic stem (ES; closed box) cells were used as controls for comparison. Values above the bars indicate relative abundance of mRNAs normalized to the expression of *gapdh* in respective cells and calibrated on undifferentiated ES cells. Different alphabet (a, b, c) on the bars indicate statistical difference (P<0.01) in respective gene expression.
(TIF)

Table S1 **Details of primer pairs used for DNA methylation analysis by bisulfite sequencing- PCR (BS-PCR).**
(DOC)

Table S2 **Details of primer pairs used for RT-PCR or real time qRT-PCR.**
(DOC)
