# Supplementary figures and images for: Differential Genomic Imprinting and Expression of Imprinted microRNAs in Testes-Derived Male Germ-Line Stem Cells in Mouse
Source: PLoS One. 2011 Jul 22;6(7):e22481. doi: 10.1371/journal.pone.0022481 (PMC3142150; doi:10.1371/journal.pone.0022481)

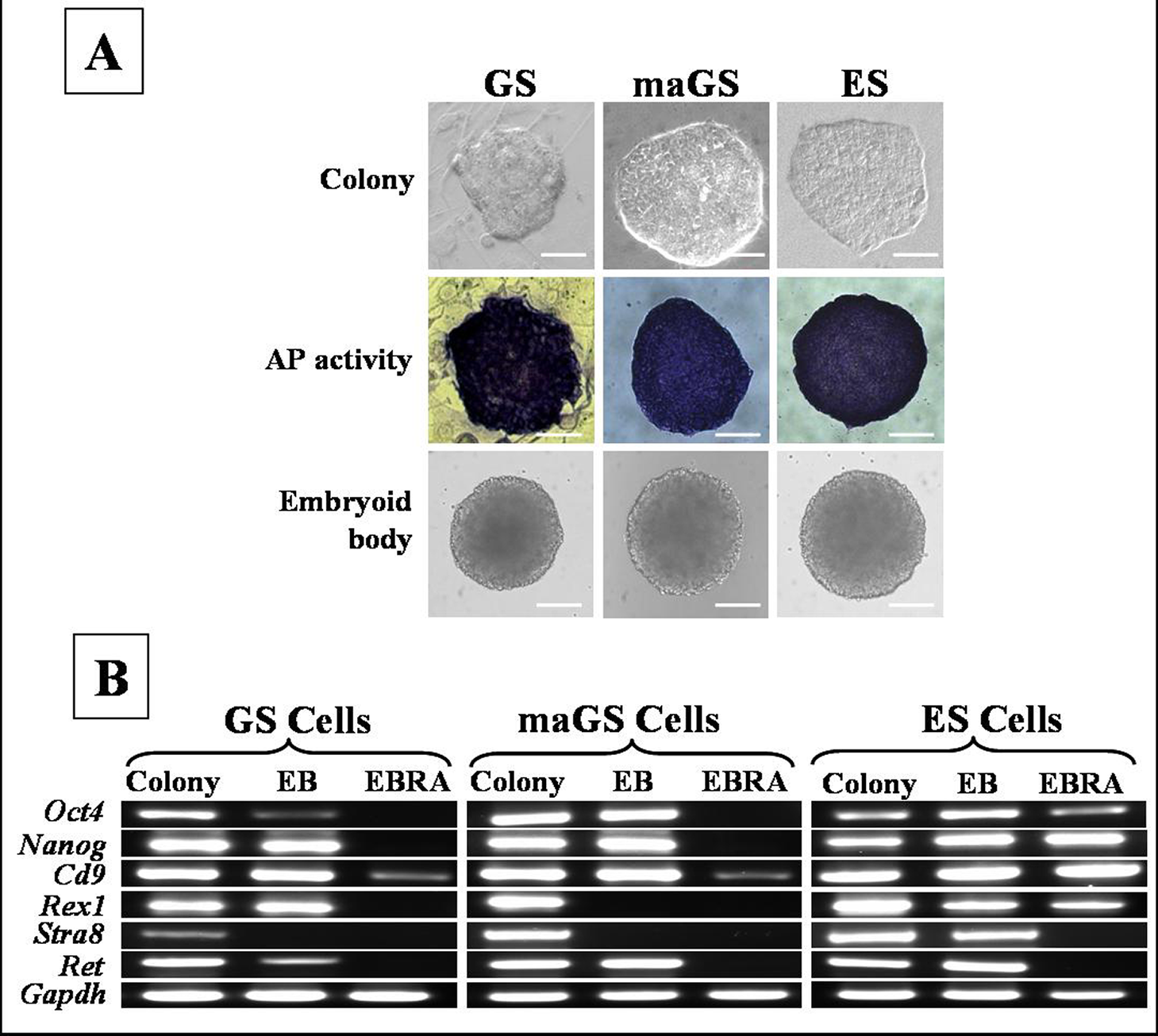

Supplement: Figure S1 — Characterization and in vitro differentiation of male germ-line (GS) and multipotent adult germ-line (maGS) stem cells in mouse. Embryonic stem (ES) cells were used as control for comparison. A: Colony characteristic, alkaline phosphatase (AP) activity and embryoid bodies generated from GS, maGS and ES cells. Calibration bar: 100 µ. B: Expression of stem cell and germ-cell marker genes in undifferentiated colonies, embryoid bodies (EB) and all-trans retinoic acid -treated embryoid bodies (EBRA) of GS, maGS and ES cells. (TIF) [file pone.0022481.s001.tif]

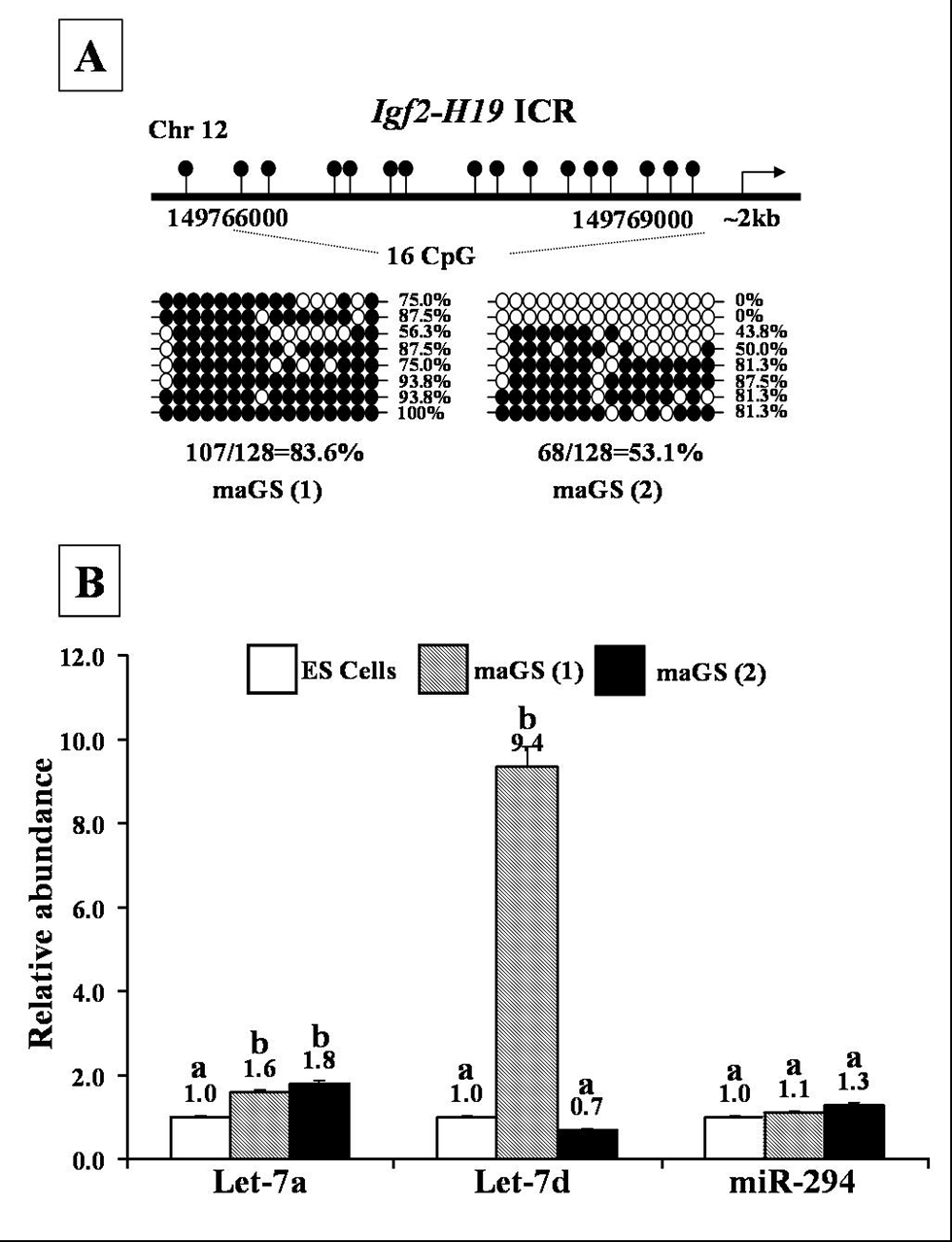

Supplement: Figure S2 — DNA methylation status of Igf2-H19 gene cluster (A) and expression of Let-7a, Let-7d and miR-194 miRNAs (B) in multipotent adult germ-line (maGS) stem cells cultured in two different culture conditions. maGS (1): maGS cells cultured in the presence of GDNF, LIF and STO feeder cell; maGS (2): maGS cells cultured in feeder-free ES cell-like culture condition. Embryonic stem (ES; open box) cells were used as controls for comparison. Values above the bars indicate relative abundance of miRNAs normalized to the expression of snoRNA in respective cells and calibrated on undifferentiated ES cells. Different alphabet (a, b, c) on the bars indicate statistical difference (P<0.01) in respective gene expression. (TIF) [file pone.0022481.s002.tif]

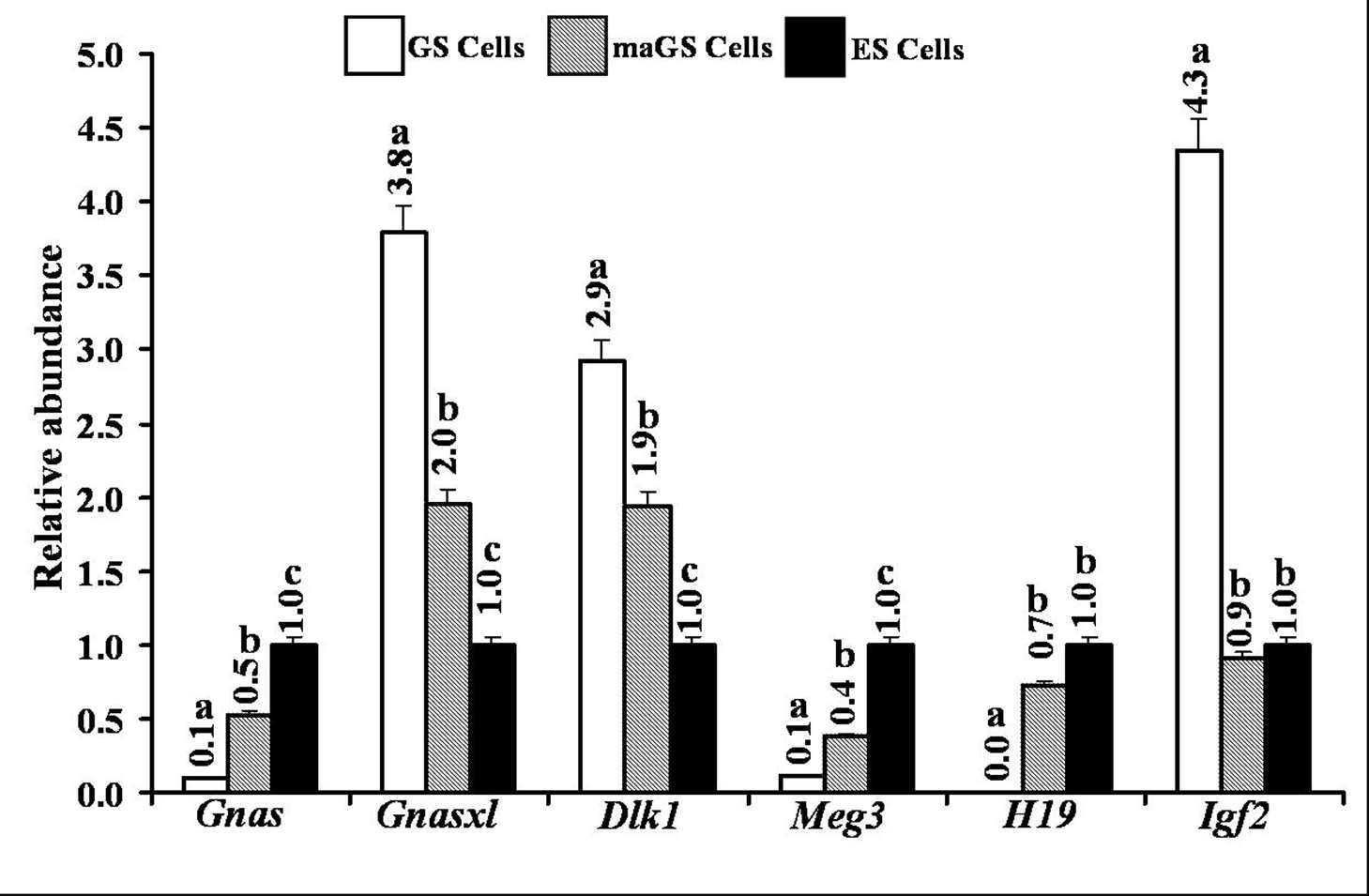

Supplement: Figure S3 — Expression of imprinted genes encoded by Gnas-Nespas ( Gnas and Gnasxl ), Dlk1-Dio3 ( Dlk1 and Meg3 ) an Igf2-H19 ( H19 and Igf2 ) gene clusters in male germ-line (GS; open box) and multipotent adult germ-line (maGS; crossed box) stem cells. Embryonic stem (ES; closed box) cells were used as controls for comparison. Values above the bars indicate relative abundance of mRNAs normalized to the expression of gapdh in respective cells and calibrated on undifferentiated ES cells. Different alphabet (a, b, c) on the bars indicate statistical difference (P<0.01) in respective gene expression. (TIF) [file pone.0022481.s003.tif]
