# Supplementary material for: Differential Genomic Imprinting and Expression of Imprinted microRNAs in Testes-Derived Male Germ-Line Stem Cells in Mouse
Source: PLoS One. 2011 Jul 22;6(7):e22481. doi: 10.1371/journal.pone.0022481 (PMC3142150; doi:10.1371/journal.pone.0022481)
Supplement: Table S1 — Details of primer pairs used for DNA methylation analysis by bisulfite sequencing- PCR (BS-PCR). (DOC) [file pone.0022481.s004.doc]

Supplementary Table S1. Details of primer pairs used for DNA methylation analysis by bisulfite sequencing- PCR (BS-PCR)

| **Gene cluster** | **Primer sequence (5’ → 3’)** | | **Annealing temperature (^o^C)** | **Amplicon size (bp)** |
| --- | --- | --- | --- | --- |
| ***Igf2-H19* ICR** | 1^st^ sense:  1^st^ antisense: | GAGTATTTAGGAGGTATAAGAATT  ATCAAAAACTAACATAAACCCCT | 50 | 422 |
|  | 2^nd^ sense:  2^nd^ antisense: | GTAAGGAGATTATGTTTATTTTTGG  CCTCATTAATCCCATAACTAT | 50 |  |
| ***Dlk1-Dio3* IG-DMR** | 1^st^ sense:  1^st^ antisense: | GTGTTAAGGTATATTATGTTAGTGTTAGG  TACAACCCTTCCCTCACTCCAAAAATT | 50 | 441 |
|  | 2^nd^ sense:  2^nd^ antisense: | ATATTATGTTAGTGTTAGGAAGGATTGTG  TACAACCCTTCCCTCACTCCAAAAATT | 50 |  |
| ***Gnas-Nespas* DMR** | 1^st^ sense:  1^st^ antisense: | GGGTGTGAGAGGTTTGGAGAGTTTATAAGA  CCAACTAAATCTCAACCACTAACCCACTCC | 62 | 607 |
|  | 2^nd^ sense:  2^nd^ antisense: | GTGTGAGAGGTTTGGAGAGTTTATAAGATT  AACTAAATCTCAACCACTAACCCACTCCCC | 62 |  |

Sequences for *Igf2-H19* ICR and *Dlk1-Dio3* IG-DMR were originally described by Nakamura et al., [1] and for *Gnas-Nespas* DMR by Kim et al., [2].

1. Nakamura T, Arai Y, Umehara H, Masuhara M, Kimura T, et al. (2007) PGC7/Stella protects against DNA demethylation in early embryogenesis. Nat Cell Biol 9: 64-71.

2. Kim JD, Kang K, Kim J (2009) YY1's role in DNA methylation of Peg3 and Xist. Nucleic Acids Res 37: 5656-5664.
