# Supplementary material for: Differential Genomic Imprinting and Expression of Imprinted microRNAs in Testes-Derived Male Germ-Line Stem Cells in Mouse
Source: PLoS One. 2011 Jul 22;6(7):e22481. doi: 10.1371/journal.pone.0022481 (PMC3142150; doi:10.1371/journal.pone.0022481)
Supplement: Table S2 — Details of primer pairs used for RT-PCR or real time qRT-PCR. (DOC) [file pone.0022481.s005.doc]

Supplementary Table S2. Details of primer pairs used for RT-PCR or real time qRT-PCR

| **Gene** | **Primer sequence (5’ → 3’)** | **GenBank accession no.** | **Annealing temp. (^o^C)** | **Amplicon size (bp)** |
| --- | --- | --- | --- | --- |
| ***Gapdh*** | CTCACTCAAGATTGTCAGCA  CATACTTGGCAGGTT | XM_001473623.1 | 58 | 326 |
| ***Oct4*** | GGCGTTCTCTTTGGAAAGGTGTTC  CTCGAACCACATCCTTCTCT | NM_013633.2 | 58 | 312 |
| ***Nanog*** | AGGGTCTGCTACTGAGATGCTCTG  CAACCACTGGTTTTTCTGCCACCG | NM_001080945.1 | 58 | 553 |
| ***Cd9*** | AGTGCATCAAATACCTGCTCTTC  CTTTAATCACCTCATCCTTGTGG | NM_007657.3 | 58 | 329 |
| ***Rex1*** | ATTTCAGAAAGGAAACCAAGGAG  CCGTTTTCTTCATTTGTTCATTC | [NM_009556.3](http://www.ncbi.nlm.nih.gov/nucleotide/157057098) | 58 | 341 |
| ***Stra8*** | GCCAGAATGTATTCCGAGAA  CTCACTCTTGTCCAGGAAAC | NM_009292.1 | 58 | 631 |
| ***Ret*** | TTAGCACAATGGAGAGATTTGGT  TCAGGGAAACATCTAATCCAACT | NM_001080780.1 | 58 | 379 |
| ***Jarid1b*** | GCTGTCCACAACCAGAGAGA  TGACAGGGAAGTTTGGTTGA | NM_152895.1 | 65 | 137 |
| ***Igf2*** | CTAAGACTTGGATCCCAGAACC  GTTCTTCTCCTTGGGTTCTTTC | NM_013633.2 | 60 | 220 |
| ***H19*** | CATGTCTGGGCCTTTGAA  TTGGCTCCAGGATGATGT | NM_001080945.1 | 60 | 245 |
| ***Meg3*** | TTGCACATTTCCTGTGGGAC  AAGCACCATGAGCCACTAGG | XR_035484.1 | 60 | 280 |
| ***Dlk1*** | CTGGCGGTCAATATCATCTTCC  GAGGAAGGGGTTCTTAGATAGCG | NM_010052.3 | 60 | 290 |
| ***Gnas*** | AGTTGGTCACCCACCATAGG  AGGGAACTTTTGTGGCCTTT | NR_003258.1 | 60 | 164 |
| ***Gnasxl*** | TGGAAGGAAAAGTCCCCTCT  GAGATCTGAAGGCGTTCCTG | NM_201617.1 | 60 | 199 |
